# Tongue Cancer Cell-Derived CCL20 Induced by Interaction With Macrophages Promotes CD163 Expression on Macrophages

**DOI:** 10.3389/fonc.2021.667174

**Published:** 2021-06-09

**Authors:** Manabu Shigeoka, Yu-ichiro Koma, Takayuki Kodama, Mari Nishio, Masaya Akashi, Hiroshi Yokozaki

**Affiliations:** ^1^ Division of Pathology, Department of Pathology, Kobe University Graduate School of Medicine, Kobe, Japan; ^2^ Division of Oral and Maxillofacial Surgery, Department of Surgery Related, Kobe University Graduate School of Medicine, Kobe, Japan

**Keywords:** tongue cancer, CCL20, cancer microenvironment, macrophage, CD163, cell–cell interaction

## Abstract

**Background:**

CD163-positive macrophages contribute to the aggressiveness of oral squamous cell carcinoma. We showed in a previous report that CD163-positive macrophages infiltrated not only to the cancer nest but also to its surrounding epithelium, depending on the presence of stromal invasion in tongue carcinogenesis. However, the role of intraepithelial macrophages in tongue carcinogenesis remains unclear. In this study, we assessed the biological behavior of intraepithelial macrophages on their interaction with cancer cells.

**Materials and Methods:**

We established the indirect coculture system (intraepithelial neoplasia model) and direct coculture system (invasive cancer model) of human monocytic leukemia cell line THP-1-derived CD163-positive macrophages with SCC25, a tongue squamous cell carcinoma (TSCC) cell line. Conditioned media (CM) harvested from these systems were analyzed using cytokine array and enzyme-linked immunosorbent assay and extracted a specific upregulated cytokine in CM from the direct coculture system (direct CM). The correlation of both this cytokine and its receptor with various clinicopathological factors were evaluated based on immunohistochemistry using clinical samples from 59 patients with TSCC. Moreover, the effect of this cytokine in direct CM on the phenotypic alterations of THP-1 was confirmed by real-time polymerase chain reaction, western blotting, immunofluorescence, and transwell migration assay.

**Results:**

It was shown that CCL20 was induced in the direct CM specifically. Interestingly, CCL20 was produced primarily in SCC25. The expression level of CCR6, which is a sole receptor of CCL20, was higher than the expression level of SCC25. Our immunohistochemical investigation showed that CCL20 and CCR6 expression was associated with lymphatic vessel invasion and the number of CD163-positive macrophages. Recombinant human CCL20 induced the CD163 expression and promoted migration of THP-1. We also confirmed that a neutralizing anti-CCL20 antibody blocked the induction of CD163 expression by direct CM in THP-1. Moreover, ERK1/2 phosphorylation was associated with the CCL20-driven induction of CD163 expression in THP-1.

**Conclusions:**

Tongue cancer cell-derived CCL20 that was induced by interaction with macrophages promotes CD163 expression on macrophages.

## Introduction

It is well known that cancer-related inflammation can promote tumor aggressiveness and that this is a target for therapeutic intervention (1). There is an inflammatory component in the microenvironment of most cancer tissue, and the significant features of cancer-related inflammation include leukocyte infiltration and prominent macrophages (1). Activated macrophages are classified into two types: tumor-suppressive M1 and progressive M2 (2–4). Macrophages in cancer tissue are called tumor-associated macrophages (TAMs) and are polarized into M2 or M1/M2 hybrid phenotype, depending on their microenvironmental factors (5). Many investigators have reported that TAM infiltration is associated with a poor prognosis in various cancers (6, 7). CD68 is recognized as a pan-macrophage marker to detect monocyte and macrophages independent of their polarization (8, 9). For the immunohistochemical detection of macrophage skewing, CD163, CD204, and CD206 are used as M2 markers (7).

Oral cancer, especially oral squamous cell carcinoma (OSCC), is a leading cause of death from oral diseases in many countries (10). The tongue is a common site of OSCC (11). Despite significant advances in cancer therapeutics over the past 30 years, the 5-year survival rate for OSCC has not changed (12). Most OSCCs occurs through oral potential malignant disorders (OPMDs), namely, premalignant lesions including epithelial dysplasia and carcinoma *in situ* (13). For the clarification of the precise mechanism of oral carcinogenesis, it is important to know the differences between intraepithelial lesion and invasive cancer.

The pathological mechanism of TAMs that contribute to OSCC progression has not been completely clarified. Numerous studies have proposed that CD163 is a TAM marker of OSCC (14–20). We showed in a previous report that subepithelial CD163-positive macrophages are associated with an immunosuppressive cytokine interleukin (IL)-10 secretion in tongue leukoplakia (TL), which is a common OPMD (21). In addition, we showed that the number of intraepithelial CD163-positive macrophages of TL with invasive carcinoma is remarkably higher than in noninvasive TL, based on immunohistochemical study using human clinical samples (22). These results suggest that the alteration of macrophage infiltrating compartment that occurred during the oral carcinogenic process may be an important therapeutic target for OSCC and their precise role should be clarified.

In this context, we investigated the interaction of macrophages and tongue cancer cells, focusing on the alteration of the macrophage infiltrating compartment. In this study, we performed a cytokine array analysis of conditioned media (CM) between macrophages and cancer cells using indirect and direct coculture of these cells to identify the cytokine that is specifically upregulated in the direct coculture system-modeled invasive tongue squamous cell carcinoma (TSCC) microenvironment. Moreover, we investigated the biological effect of this cytokine on TSCC progression by *in vitro* assays and immunohistochemical analysis using human clinical samples.

## Materials and Methods

### Patients and Tissue Samples

Tissue samples surgically resected from 59 patients with TSCC were enrolled in this study. The investigation was conducted at Kobe University Hospital (Kobe, Japan). None of the patients had received neoadjuvant chemotherapy or radiotherapy before surgery, and all patients provided their written informed consent. The study was approved by the Kobe University Institutional Review Board (No. B190043). Histological and clinicopathological evaluation was performed according to the World Health Organization’s classification of Head and Neck Tumors or the General Rules for Clinical and Pathological Studies on Oral Cancer of the Japanese Society of Oral Oncology (11, 23).

### Immunohistochemistry

All resected samples were fixed in 10% formalin and embedded in paraffin. The paraffin block specimens were cut to a thickness of 3 µm to 4 µm to prepare serial sections. We used En Vision™+ Dual Link System-HRP with 3,3′-diaminobenzidine (Dako Cytomation, Glostrup, Denmark) for immunohistochemistry. We used the antibodies to mouse monoclonal antibody against CD163 (1:100, #10D6, Novocastra, Newcastle upon Tyne, UK); rabbit polyclonal antibody against CCL20 (1:100, #ab9829, Abcam, Cambridge, UK); and rabbit monoclonal antibody against CCR6 (1:1000, #ab227036, Abcam) for primary reaction. After thorough washing with 0.05 M Tris-HCl, the sections were incubated with HRP-conjugated secondary antibodies. Before analysis, we determined the optimal conditions for immunohistochemical detection of CCL20 and CCR6 using human tonsil samples as positive controls following the manufacturer’s instructions.

To quantify CD163 expression, positive cells in whole tissues were examined in five random fields at ×200 magnification in each section. The immunoreactive intensity was scored as 1, weak; 2, moderate; or 3, strong. The percentage of positive areas was scored as 1, ≤20%; 2, 21%–50%; and 3, ≥50%. Immunohistochemical scores for CD163 expression in TSCC obtained by multiplying two parameters were divided into high and low groups as per the median value. CCL20 expression was evaluated based on the staining intensity of the whole tissue in comparison with that of corresponding normal muscle tissue. Samples with clear weaker intensity than muscle tissue were considered low, and the other samples were considered high. We assessed the CCR6 expression in the stromal area and divided it into high and low groups based on the positive area (high, >30%; low, ≤30%). Evaluation and scoring were performed by two pathologists (MS and TK).

### Cell Cultures

We used SCC25 TSCC and THP-1 human acute monocytic leukemia cell lines in this study. SCC25 is a common cell line for the OSCC research (24–28), and many researchers have used 12-*O*-tetradecanoylphorbol-13-acetate (TPA)-treated THP-1 cells as macrophage-like cells (19, 29–32). Both cell lines were obtained from the American Type Culture Collection (Manassas, VA). SCC25 cells were routinely incubated in D-MEM/Ham’s F-12 medium (Wako, Osaka, Japan) supplemented with 10% fetal bovine serum (FBS) (Sigma-Aldrich, St. Louis, MO) and 1% antibiotic–antimycotic (Invitrogen, Carlsbad, CA). THP-1 cells were incubated in RPMI-1640 (Wako) supplemented with 10% FBS (Sigma-Aldrich) and 1% antibiotic–antimycotic (Invitrogen). To induce macrophage-like differentiation, we treated THP-1 cells with 200 nM TPA (Cell Signaling Technology, Danvers, MA) as described previously (21, 33). Before performing all experiments with macrophage-like cells, we aspirated the medium and washed the wells using PBS at least three times to remove TPA sufficiently from the well.

### Coculture Assay and Preparation of CM

The indirect coculture system and the direct coculture system of macrophage-like cells with SCC25 as intraepithelial neoplasia model and invasive cancer model were established. For the indirect coculture SCC25 cells (1.0 × 10^5^ cells/well) were plated on transwell inserts with a 0.4 µm pore filter (BD Falcon, Lincoln Park, NY) in 6-well plates, and macrophage-like cells (1.0 × 10^5^ cells/well) were plated on the bottom chamber in RPMI-1640 medium with 10% FBS. For the direct coculture, SCC25 cells (1.0 × 10^5^ cells/well) were directly cocultured with macrophage-like cells (1.0 × 10^5^ cells/well) in RPMI-1640 medium with 10% FBS. After 48 h, the CM were harvested, centrifuged, and stored in aliquots at −80°C.

### Immunofluorescence

Cells were fixed with precooled methanol and subsequently incubated overnight at 4°C with primary antibodies against CCL20 (1:200, #ab9829, Abcam), mouse monoclonal antibody against CD163 (1:200, #ab156769, Abcam), rabbit polyclonal antibody against CCR6 (1:100, #ab78429, Abcam), and mouse monoclonal antibody against E-cadherin (1:400, #610181, BD Biosciences, San Jose, CA). The cells and tissues were then incubated with AlexaFluor-488^®^-conjugated donkey anti-mouse IgG secondary antibody and Cy3-conjugated donkey anti-rabbit IgG secondary antibody (Jackson ImmunoResearch Laboratories, West Grove, PA) at room temperature for 1 h. The nuclei were stained with DAPI (Wako). All images were obtained using Zeiss LSM 700 laser-scanning microscope and analyzed using the LSM software ZEN 2009 (Carl Zeiss, Oberkochen, Germany). Additionally, the tissue specimens were morphologically evaluated by hematoxylin–eosin (HE) staining.

### Quantitative Reverse Transcription-Polymerase Chain Reaction (PCR)

Total mRNA was from macrophage-like cells using an RNeasy Mini Kit (Qiagen, Hilden. Germany). Quantitative RT-PCR amplifications of *CD163* and the control gene *GAPDH* were conducted using the ABI StepOne Real-time PCR system (Applied Biosystems, Foster City, CA). Primer sequences were designed according to a previous report as follows (34): *CD163*: 5′-CGAGTTAACGCCAGTAAGG-3′/5′-GAACATGTCACGCCAGC-3′; *GAPDH*: 5′-GCACCGTCAAGGCTGAGAAC-3′/5′-TGGTGAAGACGCCAGTGGA-3′.

### Enzyme-Linked Immunosorbent Assay

The human CCL20 concentration of CM collected from coculture systems or control cells was measured using Quantikine enzyme-linked immunosorbent assay (ELISA) human CCL20 immunoassays (R&D Systems, Minneapolis, MN) as described previously (35). Briefly, the optical density of each well was determined at 450 nm and 540 nm, and the concentration was calculated using a standard curve and measured absorbance.

### Western Blotting

Cultured cells were lysed in cell lysis buffer (50 nM Tris-HCl pH 7.5, 125 mM Nacl, 5 nM EDTA, and 0.1% Triton X-100) containing both 1% protease inhibitor and 1% phosphatase inhibitor (Sigma-Aldrich, St Louis, MO). Equal amounts of protein were separated on 10% SDS-PAGE and transferred to membranes with iBlot Gel Transfer Stack (Invitrogen). The membranes were incubated with the following primary antibodies 4°C overnight: mouse monoclonal antibody against CD163 (1:100, #10D6, Novocastra); rabbit polyclonal antibody against CCR6 (1:100, #ab78429, Abcam); rabbit polyclonal antibody against ERK (1:1000, #9102, Cell Signaling Technology); rabbit polyclonal antibody against pERK (1:1000, #4370, Cell Signaling Technology); and rabbit polyclonal antibody against GAPDH (1:200, #FL-335, Santa Cruz Biotechnology, Santa Cruz, CA). The membranes were then probed with the following secondary antibodies for 90 minutes at room temperature: horseradish peroxidase-conjugated donkey anti-rabbit IgG (1:1000, #NA934V, GE Healthcare Life Sciences, Little Chalfont, UK) and anti-mouse IgG (1:1000, #NA931V, GE Healthcare Life Sciences).

### Cytokine Array

CM prepared as described above was analyzed with a Proteome Profiler Array Human XL Cytokine Array Kit (R&D Systems) according to the manufacturer’s instructions.

### Reagents

We obtained recombinant human (rh)CCL20 from R&D Systems. The antibody against CCL20 (#ab9829, Abcam) was applied to the neutralizing antibody. The MEK inhibitor U0126 (Cell Signaling Technology) was obtained for blockage of ERK phosphorylation.

### Transwell Migration Assay

Before the cell migration assay analysis, THP-1 cells (3 × 10^5^ cells/well) were treated with 200 nM TPA on the upper insert with an 8 µm pore filter (BD Falcon) to differentiate to macrophage-like cells. During the differentiation, the lower chamber was empty. For the assays, the medium in the upper insert was changed to RPMI-1640 with 1% FBS, and the insert was then exposed to the lower chamber in the presence and absence of rhCCL20 (R&D Systems). To verify the effect of CCL20 in CM, we applied CM in the lower chamber with and without CCL20 neutralizing antibody (#ab9829, Abcam) or control IgG (#ab37415, Abcam). The insert was exposed to the lower chamber for 24 h at 37°C in a CO_2_ incubator. We then gently removed the remaining cells in the upper surface of the insert with cotton swabs. The numbers of migrated cells were stained using Diff-Quik^®^ (Sysmex, Kobe, Japan). Five images at ×200 magnification were obtained from each membrane with a charge-coupled device camera, and we then counted the stained cells.

### Cell Survival Assay and Proliferation Assay

Before the assay analysis, we prepared the CM from macrophage-like cells (macrophage CM) treated with or without rhCCL20 as follows. The macrophage-like cells (5 × 10^5^ cells/well) were stimulated with rhCCL20 at 0 ng/ml, 50 ng/ml, or 100 ng/ml for 48 h, and the medium was changed to RPMI-1640 containing 0 or 1% FBS. After 48 h, the supernatants were harvested and centrifuged and stored aliquots at −80°C.

SCC25 cells (1 × 10^4^ cells/well) were seeded on 96-well plate using RPMI-1640 with serum free or 1% FBS, followed by incubation at 37°C. The cells were subsequently exposed to macrophage CMs. CellTiter^®^ 96 Aqueous One Solution Reagent (Promega) was added to each well after incubation at 37°C for 24 h. The optical density of each well was measured with a microplate reader (Infinite 200 PRO, Tecan, Mannedorf, Switzerland) at 492 nm.

### Statistical Analysis

The data from *in vitro* assays are representative results of two or three independent experiments. Data are indicated as the mean ± standard deviation (SD) or mean ± standard error of the mean (SEM). The chi-squared test was used to analyze the relationships between the patient’s clinicopathological characteristics and the immunohistochemical results. Statistical differences were evaluated by SPSS Statistics Version 22 (IBM, Chicago, IL). A value of *P* < 0.05 was considered statistically significant.

## Results

### Upregulation of CCL20 Was Specifically Induced in SCC25 Cells by Direct Coculture With Macrophage-Like Cells

**Figure 1:**
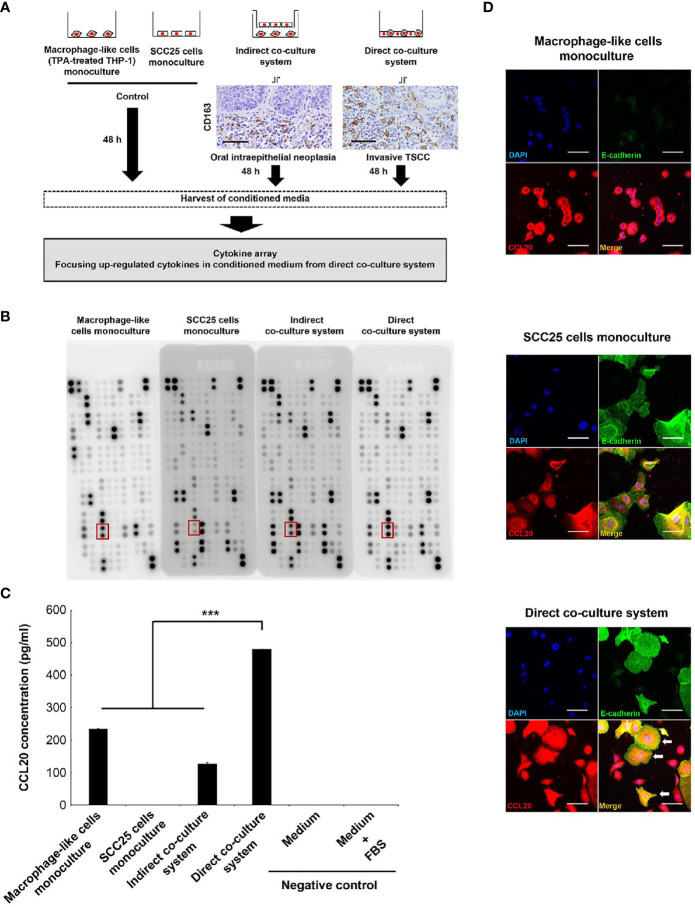
Identification of CCL20 specifically induced in SCC25 cells by close interaction with macrophage-like cells. **(A)** Workflow illustrating the strategy of cytokine array analysis. The indirect coculture system and the direct coculture system were developed to mimic an oral intraepithelial neoplasia and an invasive TSCC, respectively. Photographs indicate CD163 immunohistochemical images of each lesion. These cells were cocultured for 48 h, and CM was harvested to analyze the specifically upregulated cytokines in CM from the direct coculture system. **(B)** Photograph of membranes obtained from a cytokine array. The secretion of CCL20 was induced in CM from the direct coculture system. The red box indicates CCL20 spots. **(C)** Confirmation of the cytokine array analysis by measuring concentrations using ELISA. Significantly higher CCL20 concentration in CM from direct-culture system was confirmed compared to others. Data shown are mean ± SD (n = 5; ***P < 0.001). **(D)** Double immunofluorescence of cultured cells using CCL20 (red), E-cadherin (green), and nuclei (blue). SCC25 acquired obviously strong immunoreactivity of CCL20 by direct coculture with macrophage-like cells (arrows). Conversely, no significant change of CCL20 immunoreactivity in macrophage-like cells was observed (arrowheads). Original magnification ×200; scale bar, 50 µm.

We previously showed that a number of CD163-positive macrophages infiltrated to the surrounding cancer nest and its surrounding epithelia of invasive TSCC, whereas few these cells were observed in noninvasive lesions, and it was indicated that macrophages expressing CD163 collaborated with cancer cells more closely in invasive cancer than in intraepithelial lesions (22). Moreover, we confirmed that macrophage-like cells express CD163 by *in vitro* experiment (21). Therefore, we first established the coculture systems as shown **Figure 1A**. We conducted direct coculture of macrophage-like cells with SCC25 cells to investigate the close interactions between them in invasive TSCC. Indirect coculture was performed as a model of noninvasive lesions in which macrophages and cancer cells were separated by a basement membrane.

The significance of the interaction between tumor cells and macrophages *via* various humoral factors is well known (36–38). Thus, we thus sought to identify humoral factors specifically induced in invasive cancer and conducted a cytokine array using CM from each coculture system (**Figure 1A**). It showed higher levels of CCL20 secretion in direct CM compared with indirect coculture systems and monoculture of SCC25 cells or macrophage-like cells. ELISA confirmed that the significant increases in CCL20 concentration (**Figures 1B, C**). We subsequently assessed which cells secreted CCL20 in direct coculture systems by immunofluorescence. It was detected that high CCL20 expression was induced primarily in the cytoplasm of SCC25 cells compared with the monoculture, although the expression level in macrophage-like cells was not changed (**Figure 1D**).

### Macrophages in TSCC Microenvironment Express CCL20 Receptor

**Figure 2:**
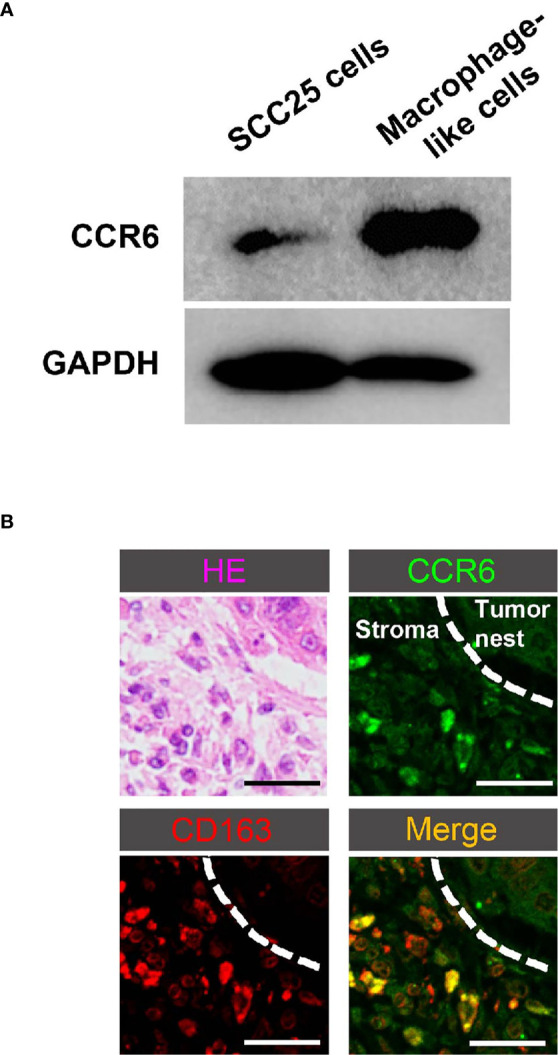
Identification of CCR6 expression in macrophages in TSCC. **(A)** Expression of CCR6 in SCC25 cells and macrophage-like cells. Western blotting showed higher levels of CCR6 expression in macrophage-like cells compared with SCC25 cells. **(B)** HE staining and double immunofluorescence of human TSCC tissue using CCR6 (green), CD163 (red), and nuclei (blue). Colocalization (yellow) of macrophages was seen in the TSCC microenvironment. Original magnification: ×200; scale bar, 20 µm.

Next, we explored the expression levels of CCR6, known as a sole receptor of CCL20 (39), in SCC25 cells and macrophage-like cells. Interestingly, it was shown that the expression level of CCR6 in macrophage-like cells was significantly higher than that in SCC25 cells (**Figure 2A**). Moreover, the expression of CCR6 was seen in part in macrophages by immunofluorescence using human TSCC samples (**Figure 2B**). Thus, we investigated the effect of CCL20 on macrophages in the TSCC microenvironment.

### High Expression Levels of CCL20 and/or CCR6 Correlate With CD163-Positive Macrophage Infiltration

**Figure 3:**
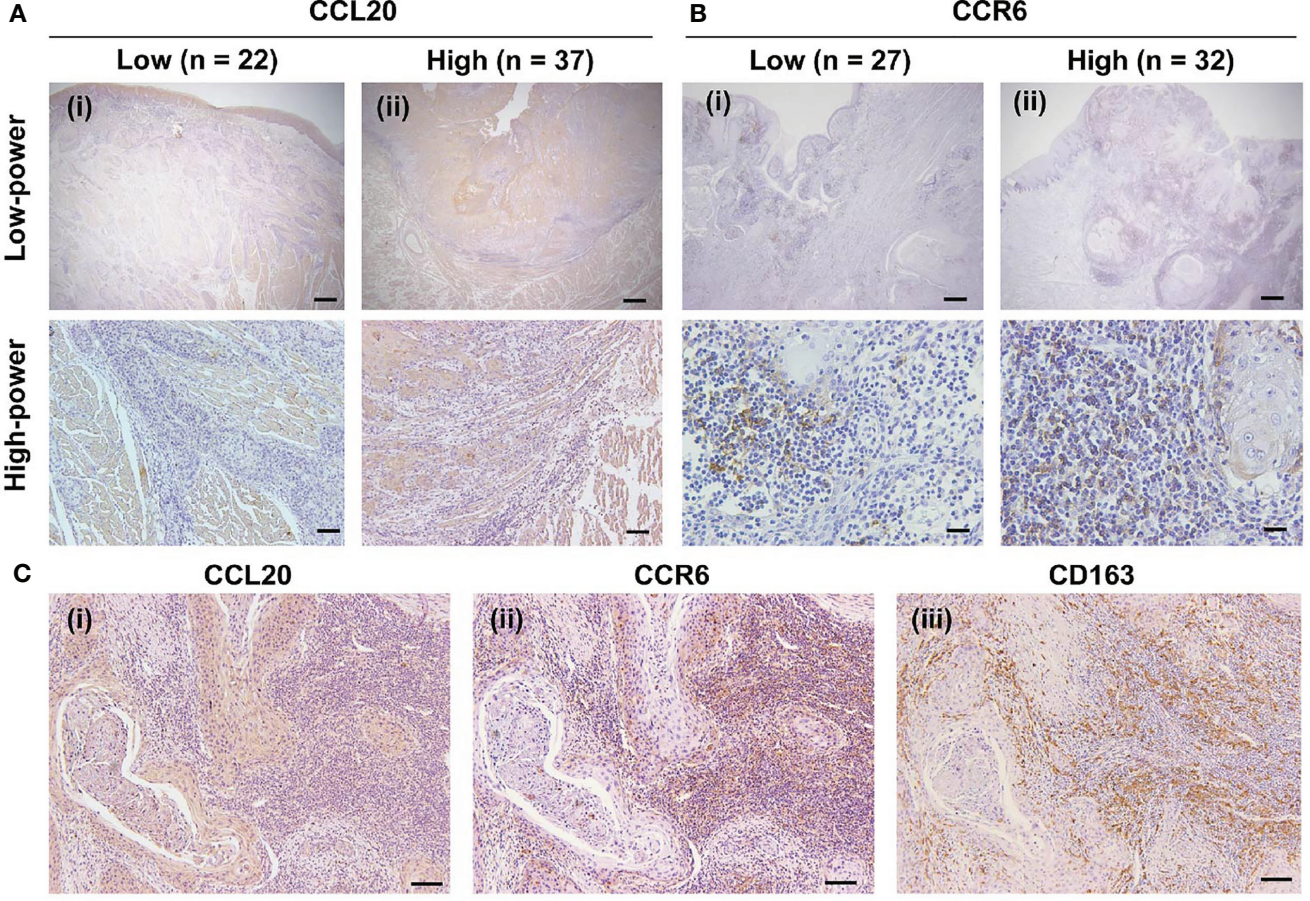
Expression of CCR20 and CCR6 in macrophages in human TSCC tissue specimens. **(A)** CCL20 immunoreaction in whole tissues was evaluated and classified into a low group (n = 22) and a high group (n = 37). Representative images of the low and high groups are shown in (i) and (ii), respectively. Original magnification ×40; scale bar, 200 µm (low-power). Original magnification ×100; scale bar, 100 µm (high-power). **(B)** CCR6 immunoreaction in the stromal area was evaluated and classified into the low group (n = 27) and the high group (n = 32) groups. The representative images for low and high groups are shown in (i) and (ii), respectively. Original magnification ×40; scale bar, 200 µm (low-power). Original magnification ×200; scale bar, 50 µm (high-power). **(C)** Representative images showing the correlation of high expression levels of CCL20 and CCR6 with the infiltration of CD163-positive macrophages expression. In the high expression level of both CCR20 (i) and CCR6 (ii), strong and diffuse immunoreactivity of CD163 were seen (iii). Original magnification ×100; scale bar, 100 µm.

We assessed whether CCL20 and/or CCR6 expression was associated with clinicopathological factors in patients with OSCC and expression of macrophage markers based on immunohistochemical analysis. We divided the CCL20, CCR6, and CD163 expressions into low and high groups as noted above. The representative immunoreactivities of CCL20 and CCR6 are shown in **Figures 3A, B**. Although CCL20 expression showed a significant positive correlation with the histological well differentiation and infiltrating CD163-positive macrophages, there was no association between CCR6 expression and any other factors. In addition, high expressions of both CCL20 and CCR6 positively correlated not only with lymphatic vessel invasion but also with CD163-positive macrophage infiltration (**Figure 3C** and **Table 1**).

**Table 1:** Relationship between CCL20 and/or CCR6 expression and clinicopathological characteristics in 59 patients with TSCC.

	n	CCL20 Expression			CCR6 Expression			CCL20 and CCR6 Expression		
		Low (n = 22)	High (n = 37)	*P*	Low (n = 27)	High (n = 32)	*P*	Non-H/H (n = 39)	H/H (n = 20)	*P*
Age		61.0	65.1	0.346	64.4	62.6	0.650	62.7	65.3	0.500
Sex										
Male	45	15	30	0.260	22	23	0.388	29	16	0.631
Female	14	7	7		5	9		10	4	
Histological grade^a^										
G1	37	10	27	0.026^*^	18	19	0.816	22	15	0.260
G2	20	12	8		8	12		16	4	
G3	2	0	2		1	1		1	1	
pT status^b^										
T1 or T2	51	18	33	0.424	23	28	0.796	34	17	0.817
T3 or T4	8	4	4		4	4		5	3	
Lymphatic invasion										
Negative	47	19	28	0.324	23	24	0.333	34	13	0.045*
Positive	12	3	9		4	8		5	7	
Vascular invasion										
Negative	41	14	27	0.451	20	21	0.483	27	14	0.950
Positive	18	8	10		7	11		12	6	
Lymph node metastasis										
Negative	32	11	21	0.614	18	14	0.078	23	9	0.308
Positive	27	11	16		9	18		16	11	
Mode of invasion (YK criteria)^c^										
1-3	33	11	22	0.479	15	18	0.957	20	13	0.315
4C or 4D	26	11	15		12	14		19	7	
CD163^+^ cells										
Low	27	15	12	0.008^**^	16	11	0.056	23	4	0.004^**^
High	32	7	25		11	21		16	16	

Data were analyzed by chi-squared test. *P < 0.05 and **P < 0.01.

^a^According to the 2^nd^ Edition of the General Rules for Clinical and Pathological Studies on Oral Cancer, G1 = well differentiated; G2 = moderately differentiated; and G3 = poorly differentiated (11).

^b^According to the World Health Organization Classification of Head and Neck Tumors, T1 = tumor ≤2 cm in greatest dimension; T2 = tumor >2 cm but ≤4 cm in greatest dimension; T3 = tumor >4 cm in greatest dimension (23).

^c^According to the 2^nd^ Edition of the General Rules for Clinical and Pathological Studies on Oral Cancer, YK-1 = well defined borderline; YK-2 = cords, less defined borderline; YK-3 = groups of cells, no distinct borderline; YK-4C = cord-like type invasion; YK-4D = widespread invasion (11).

### The Expression of CD163 and Migration in the Macrophage-Like Cells Are Promoted by Treatment With Recombinant Human CCL20

**Figure 4:**
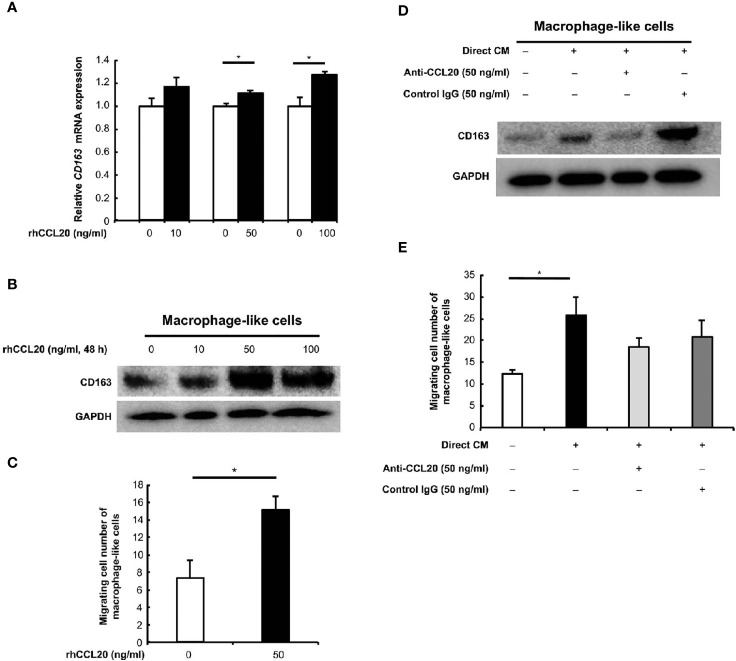
Effects of CCL20 on the macrophage-like cells. **(A)** The relative *CD163* mRNA expression was significantly induced in macrophage-like cells by rhCCL20 at 50 ng/ml and 100 ng/ml for 48 h. Data shown are mean ± SEM (n = 3; *P < 0.05). **(B)** Significant induction of CD163 expression in the macrophage-like cells was verified by western blotting. **(C)** The transwell migration of macrophage-like cells was promoted by treatment with 50 ng/ml rhCCL20 for 24 h. Data shown are mean ± SD (n = 3; *P < 0.05). **(D)** The induction of CD163 expression in macrophage-like cells by treatment with CM from direct coculture system of SCC25 cells and macrophage-like cells for 48 h, and this effect was canceled by neutralizing antibody against CCL20 antibody. **(E)** Migration of macrophage-like cells was enhanced by treatment with CM from direct coculture system of SCC25 cells and macrophage-like cells for 24 h. However, this effect tended to be suppressed by neutralizing antibody against CCL20 antibody. Data shown are mean ± SD (n = 3; *P < 0.05).

We focused on the correlation of CCL20 and CCR6 expression with CD163-positive macrophage, and we hypothesized that CCL20 was involved in CD163 expression and/or recruitment of macrophages. In vitro experiments using rhCCL20 were thus conducted to determine the effects of CCL20 on macrophages. As expected, rhCCL20 (50 ng/ml and 100 ng/ml) increased the expression of CD163 in macrophage-like cells at both the mRNA and the protein levels (**Figures 4A, B**). In addition, the migration of macrophage-like cells was induced by treatment with rhCCL20 (50 ng/ml) (**Figure 4C**). To verify the role of CCL20 from the direct coculture system in CD163 expression and migration of macrophage-like cells, these alterations of phenotypes were evaluated using direct CM with or without anti-CCL20 neutralizing antibody. Both CD163 expression and migration of macrophage-like cells were induced by direct CM. Administration of the neutralizing antibody canceled the direct CM-driven CD163 expression significantly, whereas cell migration tended to decrease (**Figures 4D, E**).

### ERK Activation by CCL20 Associates With CD163 Expression of Macrophage-Like Cells

**Figure 5:**
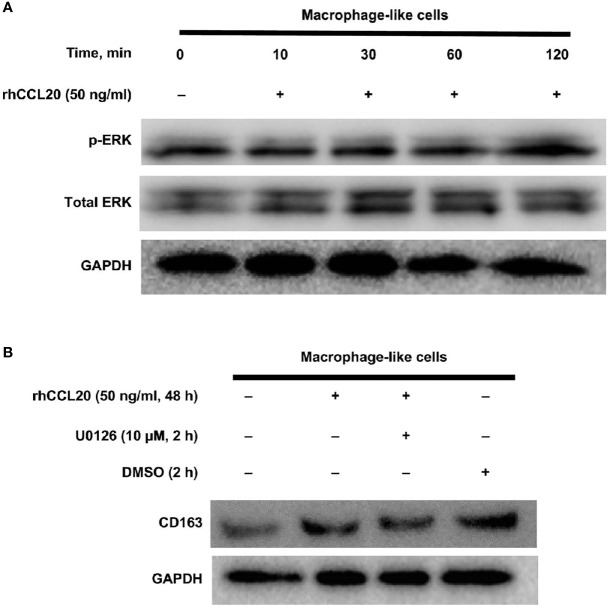
Involvement of ERK activation in the effect of rhCCL20 on macrophage-like cells. **(A)** Macrophage-like cells were stimulated by 50 ng/ml rhCCL20 for the indicated time course. The treatment for 120 minutes induced the phosphorylation of ERK. **(B)** Pretreatment of macrophage-like cells with 10 µM U0126 for 120 minutes blocked the rhCCL20 induced-CD163 expression. DMSO was added as a negative control.

Finally, the signaling pathway involved in the CD163 expression of macrophage-like cells was explored. We have previously shown that the MEK/ERK pathway is associated with the expression of CD204, a suitable M2 marker for macrophages in esophageal cancer (36). Moreover, CCL20 enhances the protumoral effects on both tumor cells and stromal cells *via* ERK phosphorylation in various cancers (40–43). Thus, we assessed whether CD163 expression of macrophage-like cells is induced through the activation of the ERK signaling by CCL20. As expected, ERK phosphorylation was increased by rhCCL20 (50 ng/ml) treatment for 2 h (**Figure 5A**). Interestingly, pretreatment with U0126 (10 µM), an ERK inhibitor, canceled the induction of CD163 expression in macrophage-like cells promoted by rhCCL20 stimulation (**Figure 5B**).

## Discussion

Chemokines are structurally associated with chemotactic cytokines and induce the migration of leukocyte not only under physiological conditions but also under inflammatory conditions (39). CCL20 was called macrophage inflammatory protein-3α, liver activation regulated chemokine or exodus-1, and CCR6 is also known as its specific receptor. Many investigators have reported the significance of CCL20–CCR6 axis in human pathogens including inflammatory and malignant diseases (39, 44). Most reports related to the CCL20–CCR6 axis in cancer progression showed their protumoral roles not only directly (such as in the proliferation, migration, and invasion of cancer cells) (41, 42, 45, 46) but also their indirect effects (such as the regulation of immune cells in the tumor microenvironment) (47–52). To the best of our knowledge, this is the first study that showed the effects of CCL20 on macrophages in human cancer tissue *via* cell-to-cell interaction between macrophages and cancer cells.

First, we clarified that CCL20 production by TSCC cells after interaction with macrophages occurs. Recently, we reported that the number of intraepithelial CD163-positive macrophages of the TL with cancer invasion is higher compared to those of TL with no cancer invasion (22). Weber et al. showed that macrophage infiltration toward the epithelium correlated with malignant transformation of oral leukoplakia (19). Their report suggests the close coexistence of macrophage and cancer cells accompanying the advance of oral carcinogenesis. Cell adhesion of macrophages and cancer cells could be pointed out as one of the important morphological alterations in the oral carcinogenic microenvironment, depending on cancer invasion. In fact, several researchers proposed the importance of direct interaction in various cancers including TSCC (17, 53–55). Conversely, we stress that the alteration of cell-to-cell interaction *via* humoral factors also should be considered, because the number of macrophages is obviously fewer than cancer cells in OSCC. Chemokines has been reported as a promising therapeutic target for patients with OSCC (56). A direct coculture system of macrophage-like cells and SCC25 cells as a model of invasive cancer microenvironment promoted CCL20 secretion specifically, whereas the secretion of no other factors was confirmed. Interestingly, the principal induction of CCL20 by direct coculture was identified in SCC25 cells by the present *in vitro* experiment. Consistent with these results, stronger immunoreactivity of CCL20 than of stromal cells was seen in human OSCC tissues by immunohistochemistry, and some studies also revealed the various involvement of CCL20 in OSCC progression in clinical samples (57–59).

The second important finding in this study is that macrophages express CCR6 in the TSCC microenvironment. The relationship between the CCL20–CCR6 axis and lymphocytes in cancer progression is well established (39, 44, 47, 48, 60–62). However, to our knowledge, no previous reports focused on macrophage expressing CCR6 in the cancer microenvironment. Because the macrophage-like cells showed markedly higher expressions of CCR6 compared with SCC25 in *in vitro* experiments, CCR6 expression in cancer cells was excluded from the immunohistochemical analysis in this study. CCR6 expression was detected in parts of macrophages of TSCC tissues by double immunofluorescence. In accordance with our hypothesis that macrophages express CCR6 in the TSCC microenvironment, the stromal CCR6 expression tended to correlate with lymph node metastasis and CD163 expression. However, it was not statistically associated with any clinicopathological factors of patients with TSCC in this study. The independent evaluation of CCR6 may not accurately reflect the role of the CCL20–CCR6 axis in OSCC. In addition, most reports of OSCC showed that CCL20 plays a protumoral role, whereas it has been reported that Tregs and Th17-expressing CCR6 play an anti-tumoral role in OSCC (63, 64). Therefore, it is reasonable to consider that the correct role of the CCL20–CCR6 axis in the human TSCC microenvironment should be determined based on the immunoreactivity for both CCL20 and CCR6.

Third, high expression of both CCL20 and CCR6 was positively correlated with the levels of CD163 expression in the human TSCC samples. To our knowledge, we assessed the correlation between the expression levels of CCL20 and macrophage markers for the first time. In addition, many investigations using human tissue samples reported that the macrophage expressing M2 macrophage marker CD163 was significantly associated with a poor prognosis (14, 15, 29, 65, 66). Furthermore, the malignant role of CD163-positive macrophages in TL was reported by some investigators (67–69). Therefore, there is sufficient evidence to support that CD163 is the most important TAM marker of TSCC. We also previously reported the CD163-positive macrophages related to the malignant behavior of oral precancerous lesions (21, 22, 70). By contrast, reliable human M1 macrophage markers for immunohistochemistry have not been established (6). Thus, we did not evaluate the correlation of other macrophage markers with CCL20 in this study. As a molecular function of CD163, it is thus considered to induce protumoral activation *via* anti-inflammatory function (6, 71). According to the results of our immunohistochemical analysis, rhCCL20 enhanced both CD163 expression and migration of macrophage-like cells. We observed a neutralizing antibody that significantly blocked CD163 expression only. However, the cell migration tended to be blocked. These results indicate that CCL20 may play a more critical role in CD163 expression than migration of macrophage-like cells. We found that the activation of ERK signaling was involved in the induction of CD163 expression of macrophage-like cells. Previous reports showed that macrophage differentiate to M2 phenotype *via* activation of signal transducer and transcription Stat3, Stat6, c-myc, or interferon regulatory factor (72–75). In the tumor microenvironment, it is well established that Stat3 activation in TAM skewing to M2 phenotype is associated with malignant behavior in various cancers (76–78). However, we did not observe the phosphorylated Stat3 in macrophage-like cells stimulated by CCL20 (data not shown). The ERK signaling may be a distinct pathway of CD163 expression in macrophage.

Finally, there are some limitations to this study. We could not elucidate how CCL20 is induced in TSCC cells by close interaction with macrophages. Several studies highlighted the proinflammatory mechanisms as inducers of CCL20. For example, a study proposed that CCL20 expression in an OSCC cell line was promoted by tumor necrosis factor-α or lipopolysaccharide (57). Another study reported that Substance P, a potent proinflammatory peptide, increased CCL20 expression of immortalized periodontal ligament cells (79). We speculate that close collaboration of cancer cells and macrophages induce some sort of unknown inflammatory alteration, which may be a trigger for the induction of CCL20 in the oral carcinogenic process. A second limitation is that we could not yet determine the precise function of macrophage stimulated by CCL20. We found that the survival and proliferation of SCC25 was promoted by CM from macrophage-like cells stimulated with rhCCL20 (**Supplementary Figure 1**). Although our supplemental data indicate that humoral factor(s) from macrophages stimulated with rhCCL20 enhance the protumoral phenotype of SCC25, this finding required additional research. Further delineation of the specific behavior of CCL20-stuimulated macrophages in OSCC tissue is a subject for future studies. Since we focused on the alteration of macrophage infiltrating compartment in oral carcinogenesis, SCC25, a pT1 TSCC cell line (24), was used in this study. However, further comprehensive analysis using more tumor cell lines should be conducted to elucidate the actual role of CCL20 in TSCC. Moreover, the number of human clinical samples in this study was relatively small. Consistent with our results that both CCL20 and CCR6 expression are also associated with lymph vessel invasion in TSCC tissue, a previous study showed that overexpression of CCL20 in patients with OSCC was associated with nodal metastasis (80). In addition, we also showed that patients with TSCC with high expression of CCL20 also had well differentiation cells. Consistent with our results, Abiko et al. found that CCL20 signals were localized primarily in the areas surrounding the cancer pearls of OSCC by *in situ* hybridization using OSCC tissues (57). Overall, these findings support the validity of our immunohistochemical analysis.

Macrophages and cancer cells acquire close interaction in association with the progression of oral carcinogenesis, and CCL20 secretion is induced in OSCC cells. More importantly, our findings present a novel concept that the CCL20–CCR6 axis may be associated with OSCC progression *via* induction of CD163 expression in macrophages.

## Data Availability Statement

The raw data supporting the conclusions of this article will be made available by the authors, without undue reservation.

## Ethics Statement

The studies involving human participants were reviewed and approved by Kobe University Institutional Review Board. The patients/participants provided their written informed consent to participate in this study. Written informed consent was obtained from the individual(s) for the publication of any potentially identifiable images or data included in this article.

## Author Contributions

MS: Acquired funding, conceived and designed the experiments, performed the experiments, analyzed and interpreted the data, and wrote the draft. Y-iK: Interpreted the data and edited the draft. TK: Prepared the tissue samples, validated the data, and performed histological evaluation. MN: Prepared the tissue samples and reviewed the clinical data. MA: Collected the clinical records and edited the draft. HY: Supervised the study. All authors contributed to the article and approved the submitted version.

## Funding

This work was supported by Grants-in-Aid for Scientific Research (19K19157) from the Japan Society of Science.

## Conflict of Interest

The authors declare that the research was conducted in the absence of any commercial or financial relationships that could be construed as a potential conflict of interest.

## Supplementary Material

Supplementary Figure 1 Macrophage-like cells stimulated with rhCCL20 contribute to the proliferation and survival of SCC25 cells. (a) The proliferative effects of SCC25 cells were enhanced by treatment with macrophage CM (prepared by stimulation with 50 ng/ml and 100 ng/ml rhCCL20) for 24 h. (b) The survival effects of SCC25 cells were enhanced by treatment with macrophage CM (prepared by stimulation with 100 ng/ml rhCCL20) for 24 h. Data are shown as mean ± SD (n = 5; **P* < 0.05, N.S. not significant).

## Abbreviations

CM, Conditioned media; ELISA, Enzyme-linked immunosorbent assay; FBS, Fetal bovine serum; HNSCC, Head and neck squamous cell carcinoma; OPMD, Oral potential malignant disorders; OSCC, Oral squamous cell carcinoma; SD, Standard deviation; SEM, Standard error of the mean; TAM, Tumor-associated macrophages; TL, Tongue leukoplakia; TSCC, Tongue squamous cell carcinoma.
