# Supplementary figures and images for: Tongue Cancer Cell-Derived CCL20 Induced by Interaction With Macrophages Promotes CD163 Expression on Macrophages
Source: Front Oncol. 2021 Jun 9;11:667174. doi: 10.3389/fonc.2021.667174 (PMC8219974; doi:10.3389/fonc.2021.667174)

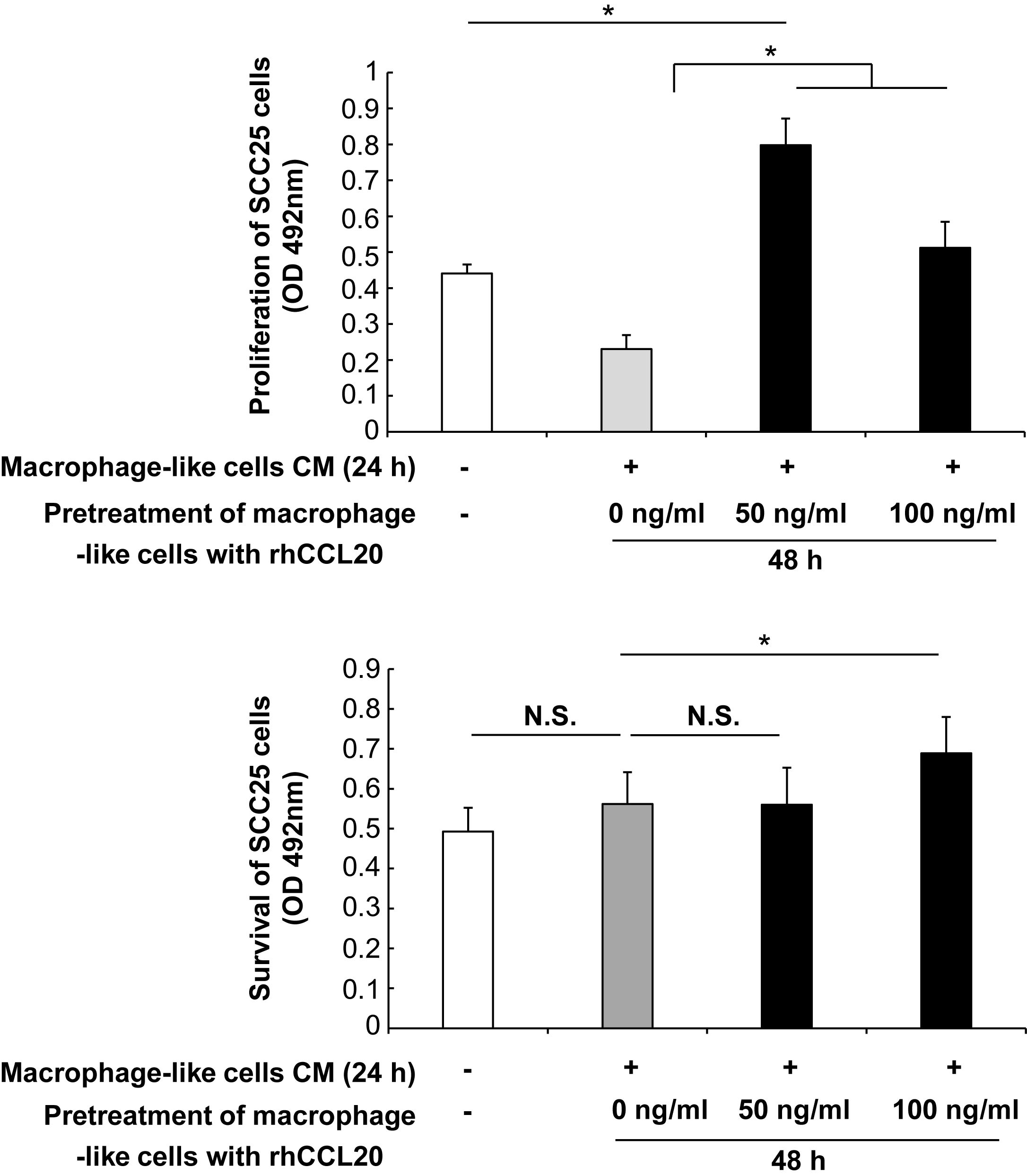

Supplement: Supplementary Figure 1 — Macrophage-like cells stimulated with rhCCL20 contribute to the proliferation and survival of SCC25 cells. (a) The proliferative effects of SCC25 cells were enhanced by treatment with macrophage CM (prepared by stimulation with 50 ng/ml and 100 ng/ml rhCCL20) for 24 h. (b) The survival effects of SCC25 cells were enhanced by treatment with macrophage CM (prepared by stimulation with 100 ng/ml rhCCL20) for 24 h. Data are shown as mean ± SD (n = 5; *P < 0.05, N.S. not significant). [file Image_1.tif]
